# Lingual and Facial Artery Fusion: A Cadaveric Report With Clinical Significance

**DOI:** 10.7759/cureus.43495

**Published:** 2023-08-14

**Authors:** Maria Piagkou, George Triantafyllou, Elena Nikolopoulou, Vasileios Karampelias, George Tsakotos

**Affiliations:** 1 Anatomy, National and Kapodistrian University of Athens, Athens, GRC

**Keywords:** anatomy, ligation, aberrant artery, trunk, fusion, superior thyroid artery, facial artery, lingual artery, variation, external carotid artery

## Abstract

The external carotid artery (ECA) anterior branches, including the superior thyroid, the lingual, and the facial artery (STA, LA, and FA) present variability among cadaveric studies. These arteries may usually originate as isolated branches from the ECA anterior surface and atypically migrate proximally or distally and/or fused into trunks with the most common fusion that of the LA with the FA, into the linguofacial trunk (LFT), and the rarer ones those of the thyrolingual and thyrolinguofacial trunks.

The current report describes a case of a bilateral fusion of the LA with the FA into an LFT and another case of a unilateral origin of the FA from the LA (aberrant FA).

In a 75-year-old donated male cadaver, a bilateral symmetrical LFT coexisted with a right-sided STA origin from the ECA proximal origin, at the level of the common carotid artery (CCA) bifurcation. In an 82-year-old donated female cadaver, at the left side, the atypical origin of the FA from the LA proximal origin coexisted with a common trunk of the left CCA with the brachiocephalic artery, and an atypical origin of the STA from the CCA, 3.65 mm inferior to the CCA bifurcation.

This report provides a detailed description of the abnormal origin of the ECA anterior branches, the potential fusion of these branches, their exact location, and the existence of an unusual origin proximal or distal to the CCA bifurcation.

Aberrant origin and course remain important in surgical and interventional approaches. A thorough understanding of the typical and variable anatomy of the ECA anterior branches ensures safe and successful intervention. Careful preoperative staging and precise dissection are essential components of this process.

## Introduction

The prevalence of the external carotid artery (ECA) variability ranges from 2.5% to 21% among cadaveric studies [1-5]. Typically, the superior thyroid, the lingual, and the facial artery (STA, LA, and FA, respectively) are referred to as the ECA anterior branches [6]. Their isolated origin was identified in 76% by Natsis et al. [7]. Usually, the STA may originate from the common carotid artery (CCA), at different levels, or at the level of its bifurcation in 61% [7]. Not unusually (24%), the STA, LA, and FA are fused into trunks [7] with the most common fusion that of the linguofacial trunk (LFT, i.e., the LA with the FA fusion), and the rarer ones, those of the thyrolingual trunk (TLT, the STA with LA fusion), and the thyrolinguofacial trunk (TLFT, the STA with LA and FA fusion), ranging from 1.4% to 3.5% [1-5] and from 0.52% to 3%, respectively [1-5, 8].

The current cadaveric report describes in detail the symmetrical (bilateral) fusion of the LA with the FA into the LFT, as well as the unilateral aberrant origin of the FA from the LA. The clinical significance of these arteries’ fusion is further described.

## Case presentation

Two body donors of Greek origin, aged 75 and 82 years, were dissected at the cervical area. The cadavers were donated to the Anatomy Department of the Medical School, of the National and Kapodistrian University of Athens, through the body donation program after written informed consent. During the two cadavers’ dissection, a bilateral LFT was identified in one cadaver, and a unilateral unusual origin of the FA from the LA, in the other.

**Figure 1:**
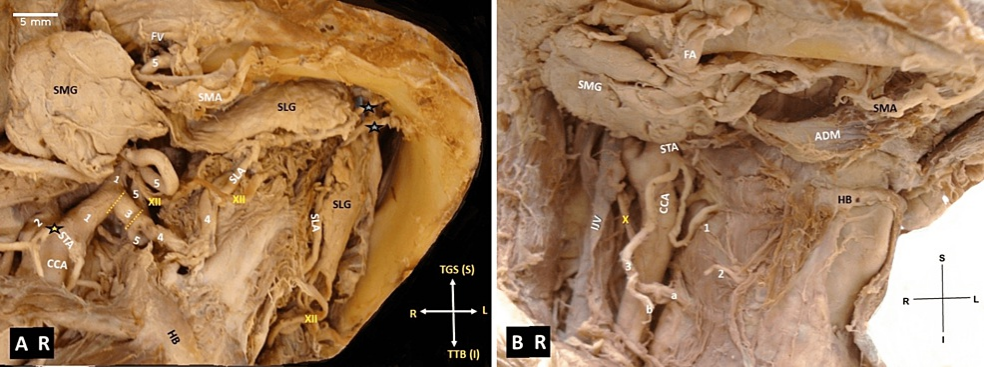
A. Right-sided (R) linguofacial trunk (3-LFT) bifurcation into lingual artery (4-LA) and facial artery (5-FA). B. Right-sided (R) superior thyroid artery (STA) division, level of the common carotid artery (CCA) bifurcation, and from the external carotid artery. A: dotted yellow line - the horizontal course of the LFT, STA - superior thyroid artery (origin from the level of the common carotid artery - CCA bifurcation), sublingual artery (SLA) course and entrance into the lingual foramina of the mandible (black-blue asterisks), ΧΙΙ- hypoglossal nerve, ICA - internal carotid artery (2), SMG - submandibular gland, SLG - sublingual gland, SMA - submental artery along the mandibular body, 1-ECA - external carotid artery and its continuation after the anterior branching division, the figure’s orientation (R - right, L - left, S - superior or TGS - to genial symphysis, I - inferior or TTB - to tongue base), HB - hyoid bone. B: STA division into the infrahyoid branch (1), the superior laryngeal branch (2) and a common stem for the anterior glandular branch (a) and the posterior glandular branch (b), X- vagus nerve, IJV - internal jugular vein, SMG - submandibular gland, FA - facial artery, ADM - anterior digastric muscle, SMA - submental artery, figure’s orientation (R - right, L - left, S - superior, I - inferior).

The bilateral LFT was identified in a 75-year-old male cadaver. At the right side, the LFT (2.9 mm in diameter at its origin) emanated 7.1 mm distally to the STA origin (level of the CCA bifurcation) and followed a horizontal course until the ECA anterior bifurcation into LA and FA. The FA followed a tortuous course (Figure 1). The LA gave off the sublingual artery, which terminated into the lingual foramina of the mandible, bilaterally, while the submental artery, branch of the FA gave off muscular branches into the anterior belly of the digastric muscle and the mental region. 

**Figure 2:**
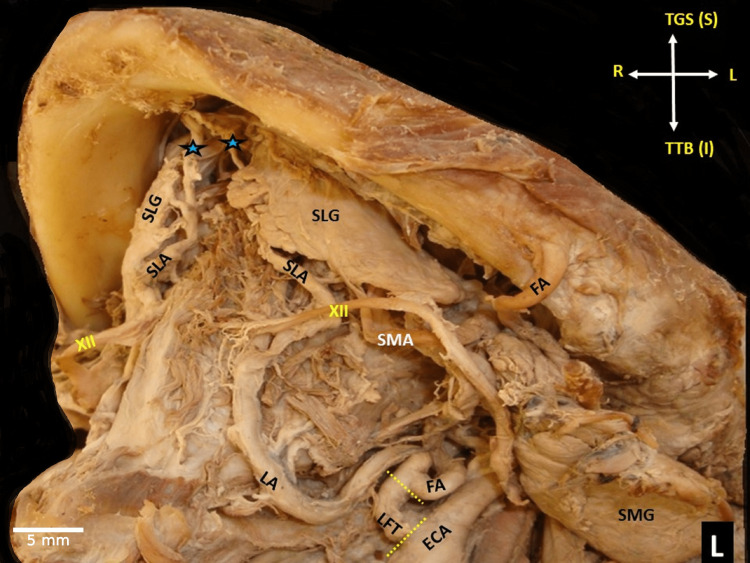
Left-sided (L) linguofacial trunk (LFT) bifurcation into the lingual and facial artery (LA and FA). SLA - sublingual artery course, and termination into the lingual foramina of the mandible (black-blue asterisks), ΧΙΙ-hypoglossal nerve, SLG - sublingual gland, SMG - submandibular gland, ECA - external carotid artery, dotted lines - the LFT ascending course, the orientation (R-right, L-left, S-superior or TGS -to genial symphysis, I - inferior or TTB - to tongue base)

The left-sided LFT (3.5 mm in diameter) originated 6.5 mm distally to the CCA bifurcation and followed an ascending course with an extreme tortuosity until the trunk division into the LA and FA (Figure 2). The CCA had a diameter of 6.12 mm on the right and 6.03 mm on the left side (level of its bifurcation). The ECA had a diameter of 4.99 mm on the right and 5.15 mm on the left side (level of its origin). 

**Figure 3:**
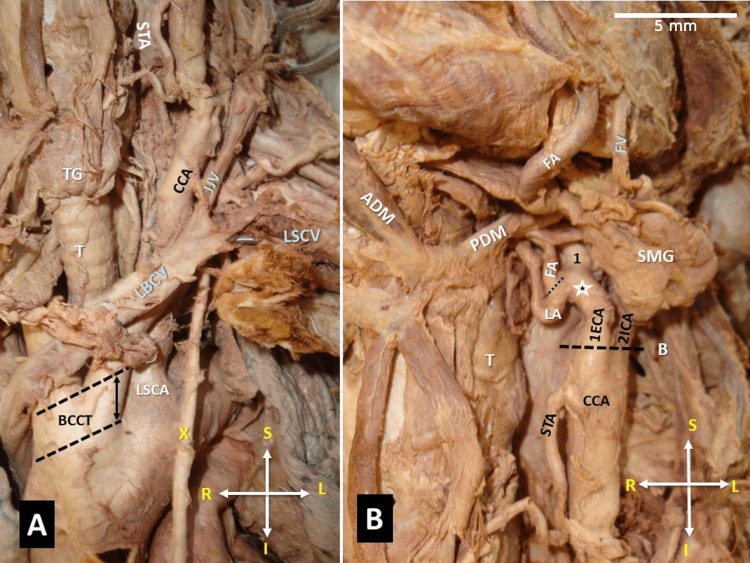
A. Common origin of the left common carotid artery (LCCA) with the brachiocephalic artery, in the form of a trunk (double arrow in between the two black dotted lines), resulting in a brachiocephalic-carotid trunk (BCCT). B. The level of origin of the lingual artery (LA) (asterisk) from the external carotid artery (ECA-1) and the proximal origin of the facial artery (FA) by the LA (line). The superior thyroid artery (STA) atypically originated from the CCA, 3.65 mm distally to the CCA bifurcation into the ECA (1) and the internal carotid artery (ICA-2) (B - black line), 1 - the ECA continuation, T-trachea, LSCA - left subclavian artery, LBCV - left brachiocephalic vein anterior course, TG - thyroid gland, LSCV - left subclavian vein, IJV - internal jugular vein, ADM - anterior digastric muscle, PDM - posterior digastric muscle, FV - facial vein, X-vagus nerve, orientation of the figure (R-right, L-left, S-superior, I-inferior)

In the second case, at the left side of the 82-year-old female donated cadaver, an atypical origin of the FA from the proximal origin of the LA was identified in coexistence with a brachiocephalico-carotid trunk (BCCT, common trunk of the left common carotid artery with the brachiocephalic artery) and an atypical origin of the STA from the CCA, 3.6 mm distally to the CCA bifurcation (Figure 3). The proximal origin of the two arteries resembled a common origin of the LA and FA from the ECA anterior surface. 

## Discussion

In the current case, a bilateral LFT was identified, distally to the STA origin, at distances (6.5 and 7.1 mm). Bilateral LFTs were identified by Fazan et al. [9] in their study in 4.8%. The prevalence of the LFT ranges between 2.7% and 31% [1-5] and the lower prevalence of the TLFT ranges from 0.52% to 3% [1-5].

During development, the common origin through a trunk is formed when the vessels from the rudimentary parts of the second aortic arch fuse together and migrate [10]. However, the exact embryologic origin of the LFT and other trunks is not known. It may be a variant of the ECA anterior branching pattern, such as the STA, the FA, or the LA.

The typical (isolated) origin for all the three anterior branches of the ECA (STA, LA, and FA) was identified in 70%-90% of the cases [9, 11] and the variant origins in 10%-30% of the cases [9, 11]. LFT was identified in 5%-20% [1-5, 9, 11], TLT in 1% [1-5, 9, 11], and the TLFT more rarely in sporadic reports arising from the ECA, or the CCA [1-5, 9]. Although in classical anatomy textbooks, the classification was extended [6] with the addition of the level or origin of the ECA anterior branches, thus concluding to six types. Whereas there is also a lack of details concerning the morphometric details of the ECA anterior branching pattern (distances between them and exact diameter), migration, and atypical origin from the ECA or the CCA surface, as isolated branches or in common trunks.

In the present study, an aberrant FA origin from the LA (resembling a common origin of both arteries from the medial surface of the ECA) was identified at the left side in combination with a BCCT and an ipsilateral atypical origin of the STA from the CCA, 3.6 mm inferior to the CCA. The aberrant origin of the STA was identified with a variable-wide prevalence ranging from 0 to 76%, while the typical STA origin was identified at 24%-100% [4, 8]. In the majority of cases, the LA and FA originated from the anterior surface of the carotid arterial axis in 76.6%, and 96.1% of the cases [12], contrariwise to Livini [13-14] who found the LA origin from the medial surface of the carotid axis in 75% and the FA origin from the anteromedial (70%) and the medial (30%) surface of the carotid arterial axis. Troupis et al. [15] identified the coexistence of a bilateral origin of an STA from the CCA and the unilateral origin of the LA from the CCA bifurcation.

Clinical significance

The in-depth knowledge of the ECA anterior branching pattern is important in catheterizations of the head and neck vessels [2], in tumor excision or embolism or anticancer drugs administration during intraarterial chemotherapy, in laryngeal, and tracheal interventions, in endo-arterial atherosclerotic plaque removal, in microsurgical arterial implantation, i.e. when evaluating the proper placement of a catheter [4]. During neck dissection for a variety of causes (thyroidectomy, parathyroidectomy, thyroglossal or brachial cysts extraction, carotid endarterectomy, aneurysms management, tracheotomy, esophageal procedures, tumor extraction, dissection of metastatic lymph nodes, or trauma management), the risk of rupture of the ECA branches is evident, in cases of variant branches, leading to extensive tissue ischemia [16]. Except for the ECA anterior branches’ variable origin, their morphometric details are clinically significant for interventional techniques [3] and their imaging is essential [17]. 

In particular, the knowledge of the anatomy of the LA is of immense importance when performing procedures on the base of the tongue [18]. Particularly, the LA and FA aberrant origin, as well as their branching pattern is of high relevance for ENT approaches and reconstructive plastic surgery [1]. The extraoral LA ligation is a high-risk maneuver, as it may lead to adjacent nerve (superior laryngeal or vagus nerve) injury, cerebrovascular accidents, and diminished vascular reserve in the ligated vessels' blood distribution. Thus, the LA origin and branching pattern should be identified, prior to the artery’s ligation, in the cervical triangles of Pirogoff, Lesser, and Beclard according to Sarna et al. study [19]. The aberrant LA and possible fusion with the other ECA anterior branches are of paramount importance for the identification of the source of uncontrolled bleeding, for the intraoral biopsy, tumor invasion, and maxillofacial trauma [2]. The knowledge of the possible symmetric fusion of the ECA branches is of high importance in bilateral neck dissection, in patients with advanced neck and head cancer with high morbidity and mortality and further complications (postoperative bleeding, edema, and chylothorax) [15]. Ischemic complications (cerebrovascular accidents and visual defects) are described after extended radical neck dissection [7]. Selective tongue or face ischemia is a possibility in case of a bilateral LFT existence. Cases of variant vessels of common origin should be managed with extreme caution due to the high risk of arterial thromboembolism and flow-related hemodynamic alterations. To help the intraoperative maneuvers in the area, Cappabianca et al. [17] highlighted the value of the use of computed tomography angiography (CTA) and magnetic resonance angiography (MRA) in characterizing the arterial variants of the cervical area.

Detailed knowledge of the variants of the ECA anterior branches, and the surgical implications they cause, careful preoperative staging, and meticulous dissection during procedures is the algorithm for a safe and successful intervention, according to Kowalczyk et al. [20]. Failure to identify the variant anatomy of an area, is a common technical error in surgical injuries, even among experienced surgeons. Thus, dissection courses and particularly detailed cadaveric reports gain teaching value, especially when focusing on the variability of the area, as well as the indications for pre-interventional imaging [21] to verify morphology [20]. Further evidence-based information is needed based on larger clinical studies with a detailed protocol to classify all possible aberrant ECA anterior branches, their migration, and/or fusion in correlation with their morphology and morphometry, thus calculating the potential risk for surgical complications, and bridging the existing gap.

## Conclusions

The present cadaveric report remains valuable as it describes in detail the aberrant origin of the ECA anterior branches, the possible fusion of two of these branches, their laterality, and the coexistence of the aberrant origin of these branches proximally or distally to the CCA bifurcation. Aberrant origin and course remain important in surgical and interventional approaches.
